# Using millimeter-wave radar to evaluate the performance of dummy models for advanced driving assistance systems test

**DOI:** 10.1038/s41598-024-52766-1

**Published:** 2024-01-27

**Authors:** Donghui Lv, Lin Yuan, Xue Bai

**Affiliations:** 1 Tianjin Sino-German University of Applied Sciences, Tianjin, 300350 China; 2 CATARC Huacheng Certification (Tianjin) Co., Ltd, Tianjin, Tianjin 300300 China

**Keywords:** Mechanical engineering, Engineering, Techniques and instrumentation, Characterization and analytical techniques, Design, synthesis and processing, Imaging techniques

## Abstract

With the rapid development of intelligent and connected vehicles, the experimental road test for the advanced driving assistance system (ADAS) is dramatically increasing around the world. Considering its high cost and hazardous situations, simulation test based on a dummy model is becoming a promising way for ADAS road test practice to reduce the experiment expanses. This study proposed a methodology for the evaluation of the performance of human and dummies with distinct designed materials based on the data extracted from the Doppler effect of millimeter-wave radar. Echo data of 8 different angles from 0 to 360 degrees, with the an interval of 45 degrees, at the same distance between the test object and the signal source is collected. Meanwhile, the echo energy is collected for correlation modeling and analysis among groups. By evaluating the performance of humans and dummies via statistical analysis, a close correlation was found which results verified the substitutability of the dummy for the ADAS experiment test. The correlation coefficient between human and dummies ranges from 0.75 to 0.93. The support vector machine (SVM) model was developed and fitted to predict the echo energy in diverse environments. The mean average error (MAE) is 5.42–11.42 in the training and testing datasets while root mean square error (RMSE) is 0.43–0.90. The methods developed in the study can simulate the real ADAS road test environment and support future experimental research.

## Introduction

Automated and connected vehicles are becoming an emerging technology for eliminating traffic congestion and safety issues^1–6^. The Advanced Driving Assistance System (ADAS) is an emerging technology for the intelligent and automated vehicles^7^. ADAS utilizes various environment perception techniques, which are installed on intelligent vehicle to promptly collect static and dynamic data of the driver as well as the surrounding environment for analysis^8^. The goal is to achieve safety and comfort by reminding the driver or actuator to intervene in the vehicle’s operation.

ADAS is an automotive system that integrates advanced sensors, processors, and software technologies to improve driving safety, comfort, and efficiency. These systems are able to provide real-time information and assistance during driving to reduce the burden on drivers, reduce the incidence of traffic accidents, and lay the foundation for the development of future autonomous driving technologies. Some common features of ADAS include adaptive cruise control, lane keeping assistance, automatic emergency braking, etc.

In order to ensure the reliability and safety of ADAS systems, effective testing methods are needed. Here are some of the key tests:1. Simulation test: The simulation environment is used to simulate various driving scenarios to evaluate the performance of the ADAS system in different scenarios. This helps to speed up the testing process and cover a wider range of use cases. 2. Field test: The ADAS system is tested in a real road environment to verify its performance in real driving conditions. This testing approach can reveal various challenges and situations that may be encountered in the real world. 3. Boundary testing: The ADAS system is tested against extreme conditions, including extreme weather, complex road conditions, and other extreme environments. This helps ensure that the system responds correctly in a variety of situations. 4. Security evaluation: Conduct security evaluation of the system, including testing the system's fault tolerance and ability to respond to attacks. This is essential to guard against potential security threats. 5. User experience test: evaluate the user experience of the ADAS system for the driver, including the friendliness of the interface and the effect of the alarm system. Make sure reminders and feedback are clear and easy to understand. 6. Software quality testing: Comprehensive quality testing is conducted for the ADAS system software, including code review, unit testing, and integration testing to ensure the stability and reliability of the software^7^.

The validation of ADAS systems ultimately necessitates field test, which in turn require the involvement of real-world environments and human participation.To ensure the safety of vehicles and humans, it is necessary to guarantee that the intelligent vehicle can effectively identify humans or other obstacles and take the initiative to avoid them during the process of driving. A comprehensive evaluation of experimental tests based on a variety of data is necessary^9–14^. Nowadays, target obstacle recognition is one of the most popular topics in the field of intelligent and connected vehicles. The on-vehicle camera has been used to detect the obstacles ahead. The advantage of cameras is that information such as obstacle type, color, etc., can be recognized by powerful pattern recognition algorithms. However, the disadvantage regarding the camera is obvious: (1) it cannot accurately measure the distance between the obstacle and the vehicle; (2) the speed of obstacle cannot be calculated; and (3) detection is highly likely to be affected by the lighting conditions. Some studies such as Xie et al.^15^ aimed at solving the problem of obstacle detection and tracking based on 3D lidars. The advantage of lidars is that they have a higher resolution of detection, while the disadvantage is that the detection can be severely affected by the weather conditions.

As a crucial part of driver assistance and automated driving systems, the radar sensor is considered to adapt to bad weather and poor lighting conditions^16^ and it is mainly applied for the automatic braking system and the intelligent cruise control system^17^. Wei et al.^18^ developed a method for front vehicle detection based on machine learning. Jing et al.^19^ described an obstacle detection method based on the Doppler feature. Nowadays, the millimeter-wave radar has been widely used in the area of intelligent transportation systems for traffic object sensing^20–22^.

There is also research focusing on improving target detection algorithms, such as the object detection and tracking algorithms study based on a millimeter-wave radar sensor which was done by Zhang et al.^23^, the human body positioning and motion state detection system study based on the radar which was conducted by Zhang et al.^24^, and the moving vehicle target classification based on the micro-Doppler effect which was proposed by Raz^25^. In addition, radar and camera sensor fusion for robust detection has been a research hotspot as a new sensing technology. Lekic and Babic^16^ proposed an automotive radar and camera fusion method using generative adversarial networks. Nabati and Qi^26^ proposed a middle-fusion approach to exploit both radar and camera data for 3D object detection after considering the problems of sensor fusion. All the previous works mainly focused on how to achieve the goal of the target recognition and active obstacle avoidance of the vehicle itself, while few people took the simulation of the test environment into consideration.

The authenticity, integrality and reliability of the road test environment significantly influence the performance of the intelligent and connected vehicle^27^. Comparing the implementation cost of real road tests, most tests adopt schemes combining software simulation, virtual-real interaction^28^ and advanced learning algorithms^29^. The environment includes roads, roadblocks, trees, houses, humans, other vehicles, motorcycles, bicycles, etc. Most environment elements could be real, except for humans who may be put in danger during the road test of ADAS.

Dummies are considered a promising way which are used as the replacement of human during the experimental tests. Field test using dummies is considered as a promising approach, with major advantages including cost reduction and increased security. Here are some key reasons to highlight these benefits:1. Testing on realistic roads requires a lot of hardware resources, including vehicles, sensor devices, and testers. These resources are quite expensive to acquire and maintain. The use of dummies can drastically reduce the need for real hardware and thus significantly reduce the cost. 2. Real road testing can take a lot of time, especially when considering the testing requirements under different driving situations and environmental conditions.Dummies can greatly shorten the testing cycle, making more frequent and comprehensive testing possible. 3. Avoiding potential hazards: There are potential safety risks associated with testing on real roads, especially when testing novel driver assistance systems. Using dummies allows testing in a simulated environment, avoiding potential hazards and accident risks. 4. Using dummies allows simulation of various emergency situations, such as traffic accidents or sensor failures, to assess the system's ability to respond in dangerous situations that are difficult to conduct on real roads. 5. Using dummies can simulate a variety of driving scenarios, including different road types, traffic conditions, and weather conditions, thus ensuring that the system works properly in a variety of situations. 6. Using dummies in field test, it is easier to control and reproduce test conditions for in-depth analysis of the system's performance in a particular context. 7. Using dummies can often be automated more easily, allowing for large-scale testing, covering more use cases, and improving test comprehensiveness and effectiveness.

In conclusion, the use of dummies for field test is a promising approach, which can reduce costs and improve safety while maintaining test quality, and provide a more flexible and controllable platform for the development and testing of advanced driver assistance systems.

However, it is still unknown whether the data from millimeter-wave radar are consistent for humans and dummies during the field test. In addition, as the echo energy is affected by multiple factors, it is of importance to develop a model of prediction in diverse environments.

This article develops a methodology for evaluating the performance of the ADAS road test of an intelligent and connected vehicle which uses dummy models to replace humans. A novel database is extracted from the Doppler effect of the millimeter-wave radar to measure the echo characteristics. The statistical correlation analysis of echo energy among humans and several dummy models is carried out to verify whether the dummies and humans are highly related in the energy of the echo wave. The support vector machine (SVM) model was developed and fitted to predict the echo energy. Findings from the study can help develop the simulation systems with a dummy for ADAS experimental road tests.

This study has made important contributions in the field of intelligent and automated vehicle experimental research, mainly focusing on the experimental analysis in this paper, the research proves that the dummy can replace the human in the development and testing of intelligent connected vehicle ADAS. Moreover, SVM and other machine learning models are used to predict the echo energy of millimeter wave radar, and obstacles can be found more accurately. Finally, the experimental design and analysis methods in this study can support larger scale ADAS testing of other types of sensors such as millimeter wave radar or radar.

## Methodology

### Doppler effect data of millimeter-wave radar

Millimeter-wave radar is generally considered an emerging technology for collecting traffic data in the Intelligent Transportation System nowadays^30,31^. It works within the frequency from 30 GHz (lower than visible light and infrared) to 300 GHz (higher than the radio) and has the advantages of both microwave guidance and photoelectric guidance. The working principle of this kind of radar is to generate the signal whose frequency increases gradually with time through the oscillator and measure the distance from the obstacle to the object according to the frequency difference between the returned waveform and the transmitted waveform. Based on the working frequency band, common vehicle-borne millimeter-wave radars include three types: 1. Short-range millimeter-wave radar (24 GHz band), which is installed in the rear bumper of the vehicle for blackspot monitoring and lane changing assistance; 2. Medium range millimeter-wave radar (76 ~ 77 GHz band), which is installed on the front bumper of the vehicle and used to detect the distance and speed of the vehicle in front, and to achieve the active safety functions such as emergency braking and automatic following; 3. Long-range millimeter-wave radar (77 GHz band). Compared with the short-range millimeter-wave radar, the resolution accuracy can be improved by 2 ~ 4 times while the accuracy of speed measurement and range measurement can also be improved by 3 ~ 5 times. It is a kind of radar sensor usually used in the advanced auxiliary driving system of intelligent and connected vehicles.

In this paper, millimeter wave radar is selected as the sensor used by the test dummies for the following reasons: 1. Adapt to different climatic conditions: millimeter wave radar performs well in most meteorological conditions, including rain, snow, fog, etc. In contrast to the performance degradation of some optical sensors (such as cameras) in bad weather conditions, millimeter wave radar is generally able to provide relatively stable performance, making them more reliable in experiments and tests. 2. High-precision distance and velocity measurement: Millimeter wave radar can provide high-resolution distance and velocity measurements, which are very important for simulating the dynamic interaction between pedestrians and cars. This makes millimeter wave radar suitable for experiments that need to accurately measure the distance and velocity of pedestrians relative to cars. 3.Simplified data processing: Because millimeter wave radar provides parameters such as range and velocity directly, it may be easier to process data than some sensors such as cameras. The echo energy calculated by millimeter-wave radar measurement better reflects the correlation between real and dummy, which helps to reduce the computational complexity and improve the efficiency of the experiment^16,17^.

**Figure 1:**
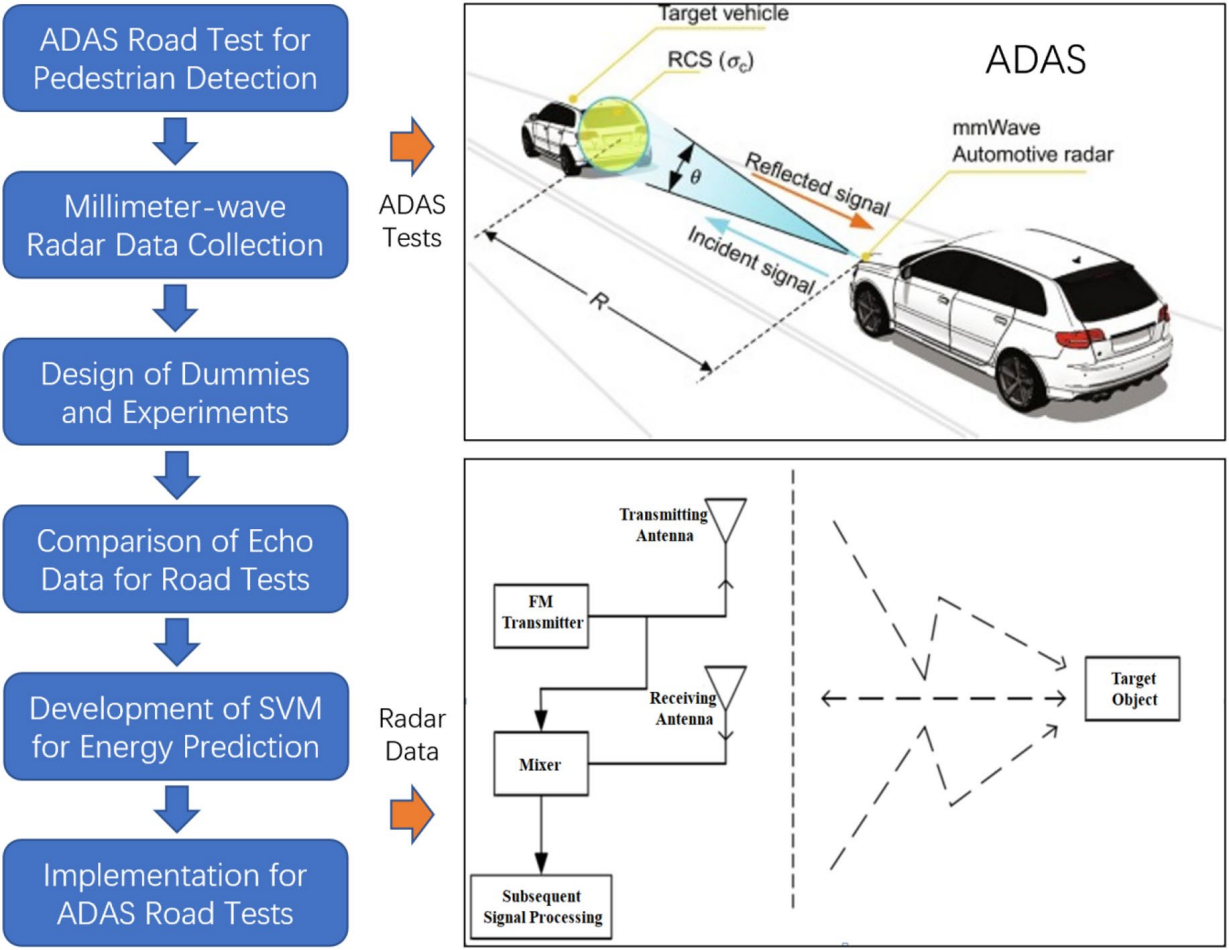
Research framework and principles of millimeter-wave radar.

The millimeter-wave radar transmits the modulated electromagnetic wave signal through the transmitting antenna while the sensor receives the reflected electromagnetic wave signal at the antenna end. After the radio frequency front-end circuit processing, the radar system will carry out the relevant signal processing on the reflected signal of the target, and then parameters such as the distance, velocity and azimuth of the target can be calculated. The schematic diagram of radar target detection is shown in Fig. 1.

**Figure 2:**
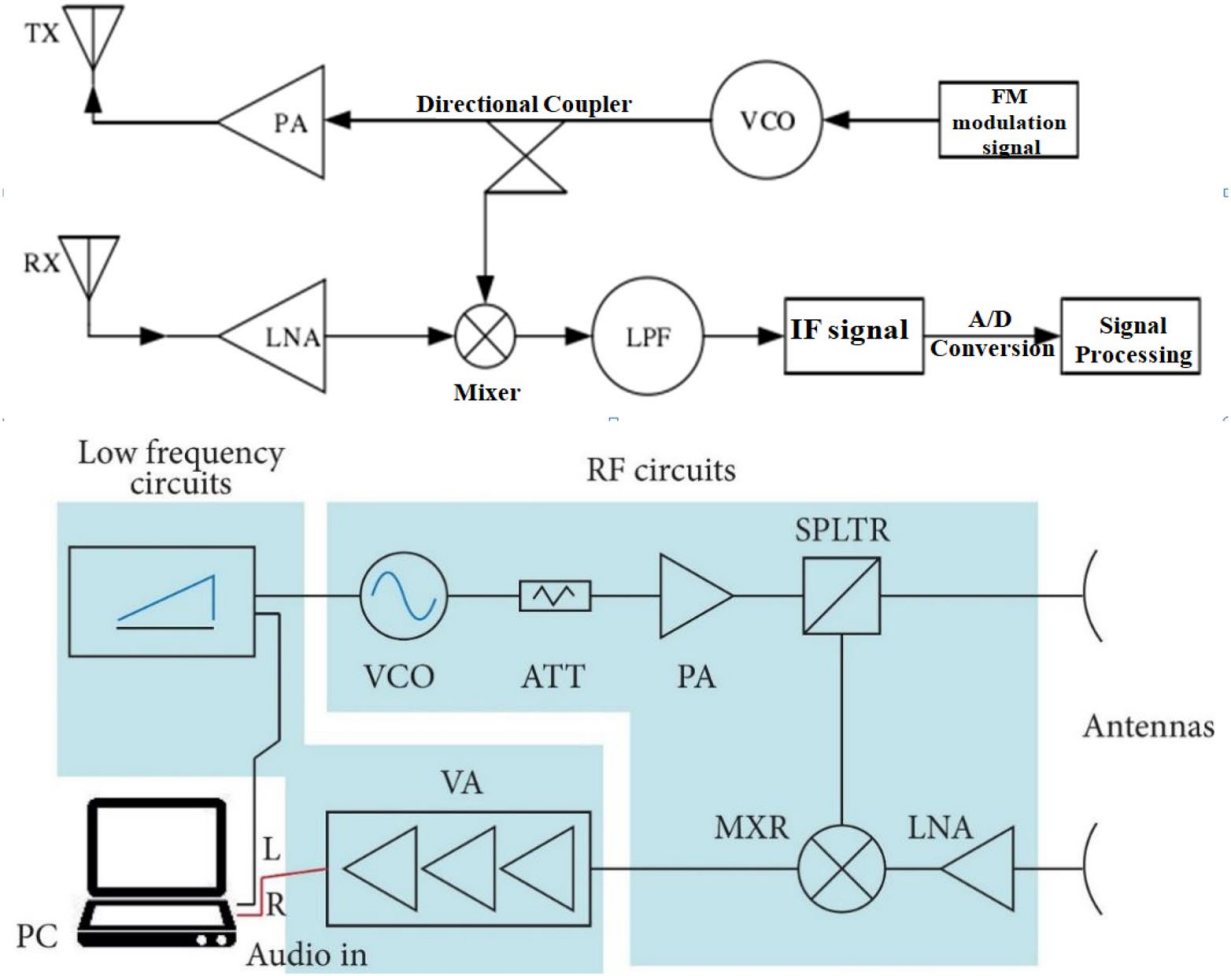
Radar radio frequency schematic diagram.

The radar sensor signal is generated by the frequency generator, and then the frequency synthesizer would combine signals together. The high-frequency signal is amplified and transmitted by power amplifier (PA). The other part is transmitted to the mixer through a directional coupler, waiting for consorting with the received signal. When the transmitted signal which propagates through the air meets the target object in the detection area, the signal is reflected back and caught by the receiving antenna. As the received signal is relatively weak, a low noise amplifier (LNA) is used to enlarge the signal, and the amplified signal is combined with the mixer to get an intermediate-frequency signal to adjust the amplitude and phase of the intermediate-frequency signal. The intermediate-frequency signal is converted into a digital signal after the A/D conversion. The speed, angle and distance of the object can be obtained after processing by DSP and related algorithms. The principle of radar RF is shown in Fig. 2^19^. In Fig. 2,VOC means Voltage-Controlled Oscillator;PA means Power Amplifier;TX means Transmitter; RX means Receiver; LPF means Low-Pass Filter;ATT means Attenuator;SPLTR means Splitter;LNA means Low-Noise Amplifier;VA means Voltage Amplifier;VA MXR means Mixer.

### Theoretical basis of experimental development

Millimeter-wave radar uses Doppler frequency to obtain the radial velocity and range change rate of the object, to distinguish dynamic and static objects. The Doppler phenomenon describes the center frequency shift of an incident wave caused by the target relative radiation signal source along with the motion of the object. The frequency shift can be positive or negative depending on the direction of the target motion. The waveform incident on the target phase wave was separated by wavelength equally. Approaching the target or approaching the millimeter-wave radar will make the phase wave of the reflected wave closer to each other and force the wavelength to get smaller. Away from the target or away from the millimeter-wave radar, will make the reflected phase wave spread forward and the wavelength longer.

Assume that the target approaches the radar at a radial velocity $v_{{0}}$(the velocity component of the target along the radar line of sight). Taking $R$ as the reference range and $t$ as the reference timing, the object target distance range at any time *t* can be expressed by the equation:

1 $$ {\text{R}}\left( t \right) = R - v_{0} t $$

Suppose that the signal transmitted by millimeter-wave radar is:

2 $$ {\text{X}}\left( t \right) = A\cos (2\pi f_{0} t) $$

The center frequency of millimeter-wave radar is represented by $f_{0}$, so the received signal of millimeter-wave radar can be expressed as following equations:

3 $$ {\text{X}}_{{\text{r}}} (t) = X[t - \varphi (t)] $$

where $$\phi \left( {\text{t}} \right)$$ is:

4 $$ \phi \left( {\text{t}} \right) = \frac{{2}}{{\text{c}}}\left( {R_{{0}} { - }vt} \right) $$

Substituting the above formulas (2) and (4) into formula (3), formula (5) can be obtained as:

5 $$ X_{r} \left( t \right) = A_{r} cos\left[ {2\pi \left( {f_{0} t - f_{0} \frac{{2R_{0} }}{c} + \frac{{2f_{0} vt}}{c}} \right)} \right] $$

where $A_{r}$ is a constant and the phase:

6 $$ \psi_{{0}} = {2}\pi f_{0} \frac{{2R_{0} }}{c} $$

The energy of a signal in a certain period can be obtained by integrating the signal in time. However, when it comes to a large signal $X\left( t \right)$, as the positive and negative areas of the signal may offset each other and behave like a small-scale signal, the signal size is defined as the area below $X^{2} \left( t \right)$, for this value is always positive. The energy of the signal will be gained as follows^19^:

7 $$ {\text{E}}_{{\text{x}}} = \int\limits_{ - \infty }^{\infty } {\left| {{\text{X}}\left( t \right)} \right|}^{2} dt $$

By calculating the echo energy of the transmitted signal encountering obstacles, the correlation between the echo energy can be computed through correlation analysis.

Correlation coefficient is a statistical analysis index to judge the direction and degree of linear correlation, which is calculated by the product ratio of the standard deviation of two variables.

8 $$ r = \frac{{\sigma_{xy}^{2} }}{{\sigma_{x} \sigma_{y} }} = \frac{{n\sum {xy - \sum {x\sum y } } }}{{\sqrt {n\sum {x^{2} - \left( {\sum x } \right)^{2} } } \sqrt {n\sum {y^{2} - \left( {\sum y } \right)^{2} } } }} $$

The range of correlation coefficient $r$ is: $- 1 \le r \le 1$. While $r > 0$, two quantities are positively correlated, vice versa two quantities are negatively correlated; $r = 0$ means two quantities are not correlated; $\left| r \right| = {1}$ means two quantities are completely linearly correlated; $\left| r \right| > {0}{\text{.8}}$ means two quantities are highly linearly correlated; $0.5 < \left| r \right| \le 0.8$ means two quantities are significant linearly correlated; $0.{3} < \left| r \right| \le 0.{5}$ means two quantities are low linearly correlated; $\left| r \right| \le {0}{\text{.3}}$ means two quantities are unrelated.

### SVM for prediction

There are many algorithms for prediction tasks. Among them, the machine learning models, which does not require mathematical assumptions about the data structure, are found to have better robust performance in prediction.

There are many machine learning models such as Random forest、CatBoost、lightGBM and the SVM method^33^. Running time is one of the important indexes to measure the time complexity of an algorithm. The SVM model is one of them which has been successfully applied in much research for a variety of prediction studies. In this study, random forest, CatBoost, lightGBM and other methods were used to compare with the SVM method used in this study. The running time of each method is counted when it achieves a similar average error and root mean square error. All experiments in this section are run under the Matlab 2022b Win11 64-bit version environment, the running computer processor is an Inter(R) Core i5-1155G7, and the running memory is 16.0 GB. The running time of different methods can be seen in Table 1.

**Table 1:** Running time of different methods.

Methods	Random forest	CatBoost	lightGBM	SVM
Running time (s)	1.89	1.78	1.45	1.23

Table 1 shows that the SVM method selected in this study has the least running time and smaller time complexity compared with Random forest, CatBoost, and lightGBM.

The SVM model is used in this study to predict the echo energy of millimeter-wave radar. The SVM is developed for solving classification and prediction tasks^32^. In particular, SVM model maps the input vector,** X**, into a high dimensional feature space. An optimal separating hyperplane is drawn in this higher dimensional space to separate the data into different groups, while maximizing the margin between the linear decision boundaries. In SVM the input is defined as vectors ***x***_*i*_ $\in$ *R*^*n*^ for *i* = 1,2,…,*N*, which represent the full set of echo energy-related variables, and the training output is defined as ***y***_*i*_ $\in$ *R*^*n*^. The hyperplane for separating outcomes can be written as the set of points **X**:

9 $$ {\mathbf{W}} \cdot {\mathbf{X}} - b = 0 $$

where “∙” denotes the dot product and the vector **W** is a normal vector. In two-category classification, given a training set of instance-label pairs (*x*_*i*_, *y*_*i*_), the SVM model needs to solve the optimization problem^29^:

10 $$ \begin{gathered} \;\;\;\;\;\;\;\;\mathop {\min }\limits_{w,b,\xi } \;\;\;\;\;\;\;\tfrac{1}{2}w^{T} w + C\sum\limits_{i = 1}^{N} {\xi_{i} } \hfill \\ {\text{Subject}}\;{\text{to}}\;\;\;\;\;\;y_{i} (w^{T} \phi (x_{i} ) + b) \ge 1 - \xi_{i} , \hfill \\ \;\;\;\;\;\;\;\;\;\;\;\;\;\;\;\;\;\;\;\;\xi_{i} \ge 0. \hfill \\ \end{gathered} $$

where *ξ* are slack variables measuring the misclassification errors, and *C* is the penalty factor to errors introducing additional capacity control within the classifier. The constraint along with the objective of minimizing function can be solved using Lagrange multipliers. Several kernels have been proposed by researchers, and the most used radial basis function (RBF) was used for crash injury severity prediction in this study.

The version of SVM for regression was proposed by Drucker et al.^33^ which is also called support-vector regression (SVR). The model produced by support-vector classification (as described above) depends only on a subset of the training data. This is due to the fact that the cost function for building the model does not care about training points that lie beyond the margin. The model produced by SVR depends only on a subset of the training data, as the cost function for building the model ignores any training data close to the model prediction. The input of the SVR model is the experimental factors such as dummy type, angle, etc., while the output is the echo energy. The results are given in the following section.

### Institutional review board statement

All subjects were informed of the purpose of the study and all consented in writing to be included in the study. The Ethical Committee of Tianjin Sino-German University of Applied Sciences Hospital approved the research protocol in accordance with the ethical guidelines of the World Medical Association (Declaration of Helsinki).

### Informed consent statement

This study confirms that informed consent has been obtained from all subjects and/or their legal guardians.

## Experiment tests

### Design and development of dummy

**Figure 3:**
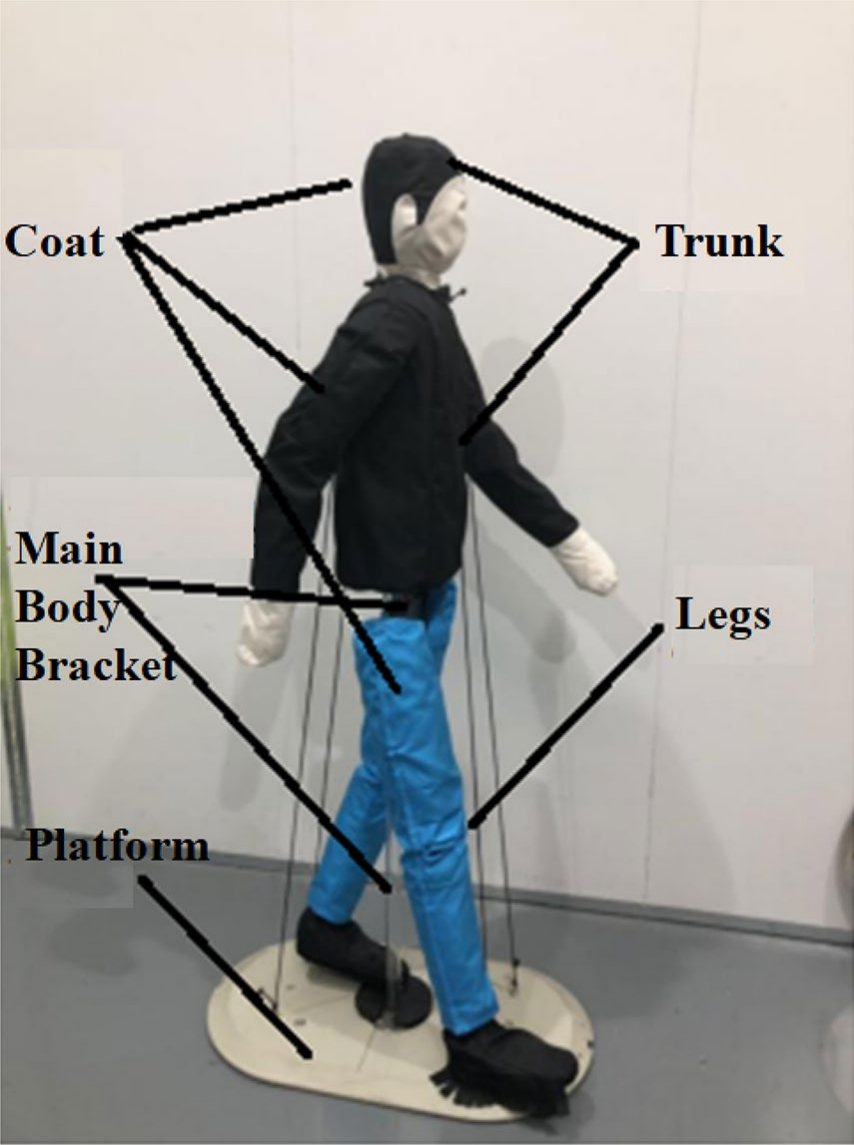
ADAS road test dummy.

The dummy model is used to replace humans during the ADAS road test. The model consists of a trunk, leg, main body bracket, coat and connector. The trunk and legs are filled with the microwave-absorbing material (Type 1 dummy) to effectively reduce the influence of the metal components on the radar wave and create a good electromagnetic environment for the echo. The EPE material (Type 2 dummy) is also used in the trunk and legs to simulate the state of the human body. The coat uses Oxford fabric to simulate the real clothing material; the main bracket adopts the φ35*3 PC tube, and the PC material can reduce the influence of the electromagnetic wave echo. The supporting platform supported is made of steel plate, which increases the overall weight of the dummy and moves the center of gravity lower for the purpose of making it hard to fall during the test. The surface of the platform is painted, which can also reduce the interference of the electromagnetic wave echo. The dummy model developed in our study is shown in Fig. 3 and Table 1.

### ADAS road test experiment

The main content of the experiment is the target contrast test based on automobile millimeter-wave radar. The purpose of this test is to verify the consistency of electromagnetic characteristics between humans and dummies. The echo data of humans and dummies at the same distance but at different angles is collected by 77 GHz millimeter-wave radar. The Radar Range-Doppler image is obtained by signal processing method. The statistical characteristics of humans and dummies are analyzed to make a comparison of the electromagnetic characteristics among them.

**Figure 4:**
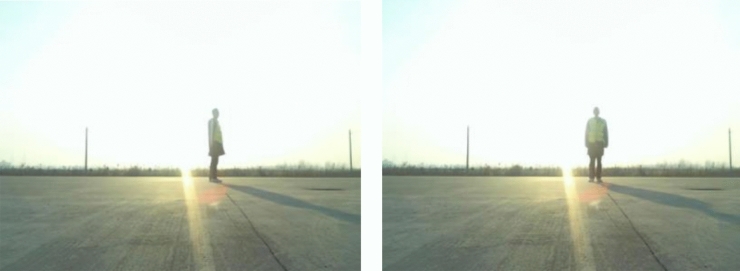
Example of 0-degree position and 90-degree position for ADAS test.

To compare the data features, two types of dummies with microwave-absorbing materials and filling materials are simulated according to a human with a height of 175 mm and a weight of 75 kg. For the comparison experiment, an adult man with the same height and weight was selected. According to the international standard for ADAS testing, the distance between the experimental vehicle and the target was set at 15 m. This is the distance for a vehicle decelerating from 60 km/h to full stop. The experimental vehicle and the target are stationary while the angle of the target is changing constantly. The set of test angles under the range from 0 to 360 degrees with an interval of 45 degrees. 0 degree is defined as the right side of the test dummy or human facing the vehicle while the left and right sides of the test target are parallel to the longitudinal direction of the vehicle. The test target is on the center line of the vehicle, as shown in Fig. 4. In each designed scenario, the test was conducted for 10 times repeatedly. During each test, the speed of the target, the range of the target, the angle of the target and the reflection cross section (RCS) was collected for 30 s for further analysis. The RCS information of the target is determined by the Doppler effect of the radar, and the echo energy is obtained by integrating the RCS through the software.The Ethical Committee of Tianjin Sino-German University of Applied Sciences Hospital approved the research protocol in accordance with the ethical guidelines of the World Medical Association (Declaration of Helsinki). This study confirms that informed consent has been obtained from all subjects and/or their legal guardians.

The experimental test involved the collection of 360-degree test data.360-degree data collection is crucial in sensor and system evaluation, especially for safe driver assistance systems or autonomous driving systems in vehicles. Here are some explanations on why 360-degree data collection was chosen and how it contributes to a thorough assessment:

1. Panoramic view: 360-degree data collection means that sensors capture a complete view of their surroundings at the same time. For vehicle safety systems, knowing the full range of environmental information is essential to accurately identify and predict possible obstacles, pedestrians, vehicles, or other important objects.
2. Comprehensive assessment: Collecting 360-degree data allows the system to fully assess the surrounding environment and better understand the actual situation of the vehicle. This helps the system to make decisions and react more accurately, improving driving safety.
3. Avoid blind spots: 360-degree data collection can help avoid the existence of blind spots. Data collection by sensors at different angles and directions can fill the blind areas in all directions, thus reducing the possible unrecognized areas.
4. Importance of collecting data from multiple angles and distances: Data collection from different angles and distances is crucial for gaining insight into the surrounding environment. Data from different angles can provide different perspectives and information, for example, close data can help identify surrounding obstacles close to the vehicle, while long distance data can help detect distant obstacles or traffic situations. This multi-angle and distance data collection can also help the system to better perform object tracking and prediction. Compared with only focusing on data from a single direction or a specific distance, omnidirectional data collection can provide more comprehensive and accurate knowledge of the environment and help the system to make more reliable decisions.

In summary, 360-degree data collection is to ensure that the vehicle's safety driver assistance system or autonomous driving system has a comprehensive understanding of the surrounding environment and can better perceive, identify and respond to various situations on the road. The collection of data from multiple angles and distances is essential for the comprehensive evaluation and accurate judgment of the system, helping to improve the safety and reliability of vehicle driving.

## Results of data analysis

**Figure 5:**
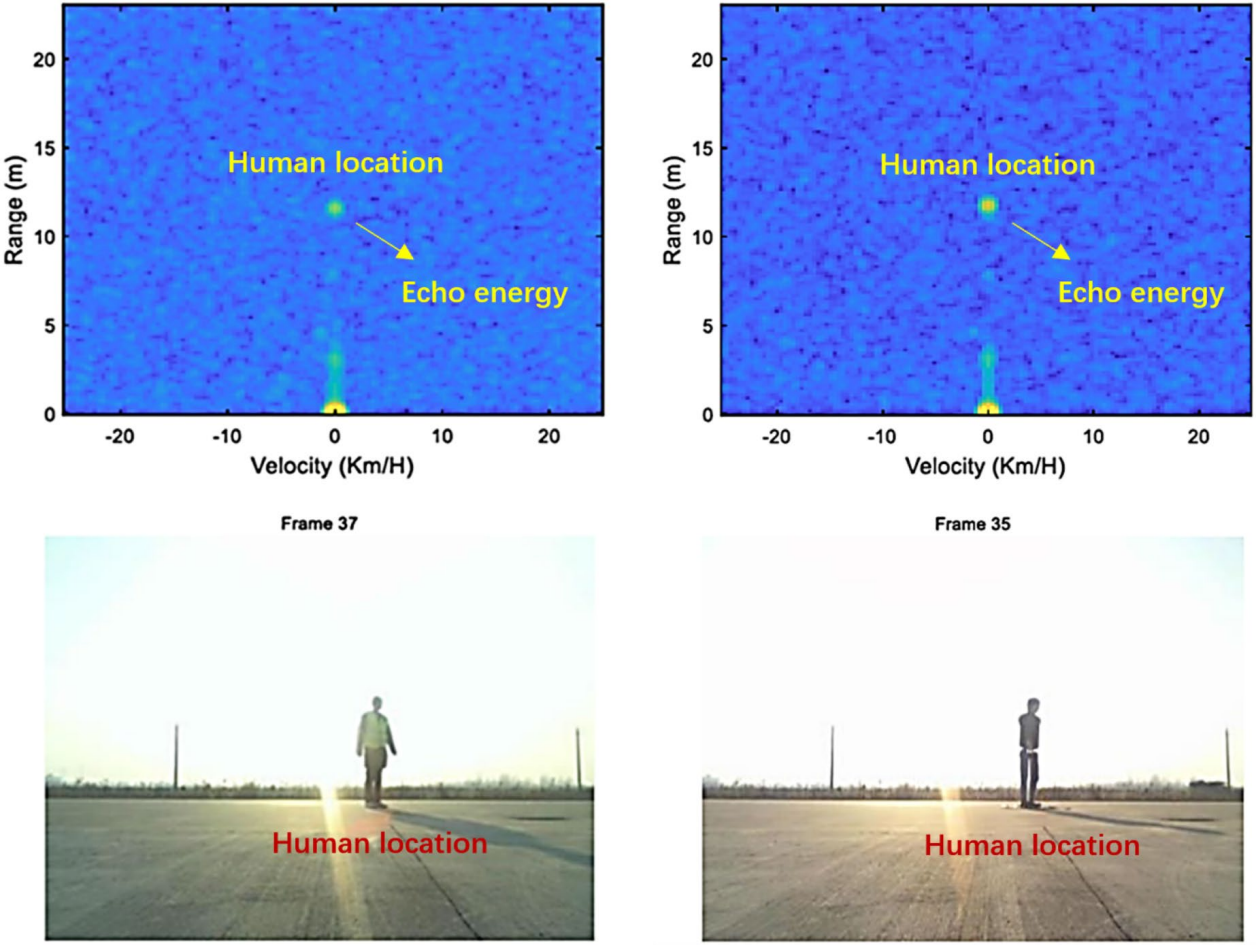
Illustration of Range Doppler map of human and dummy test.

The echo data of humans and dummies was collected separately during the test. The statistical characteristics of the radar range Doppler map are analyzed, as shown in Fig. 5. It can be concluded that both humans and dummies have effective echo characteristics as the imaging is clear on the Doppler picture, and the performance of the electromagnetic wave echo can be conjectured.

**Figure 6:**
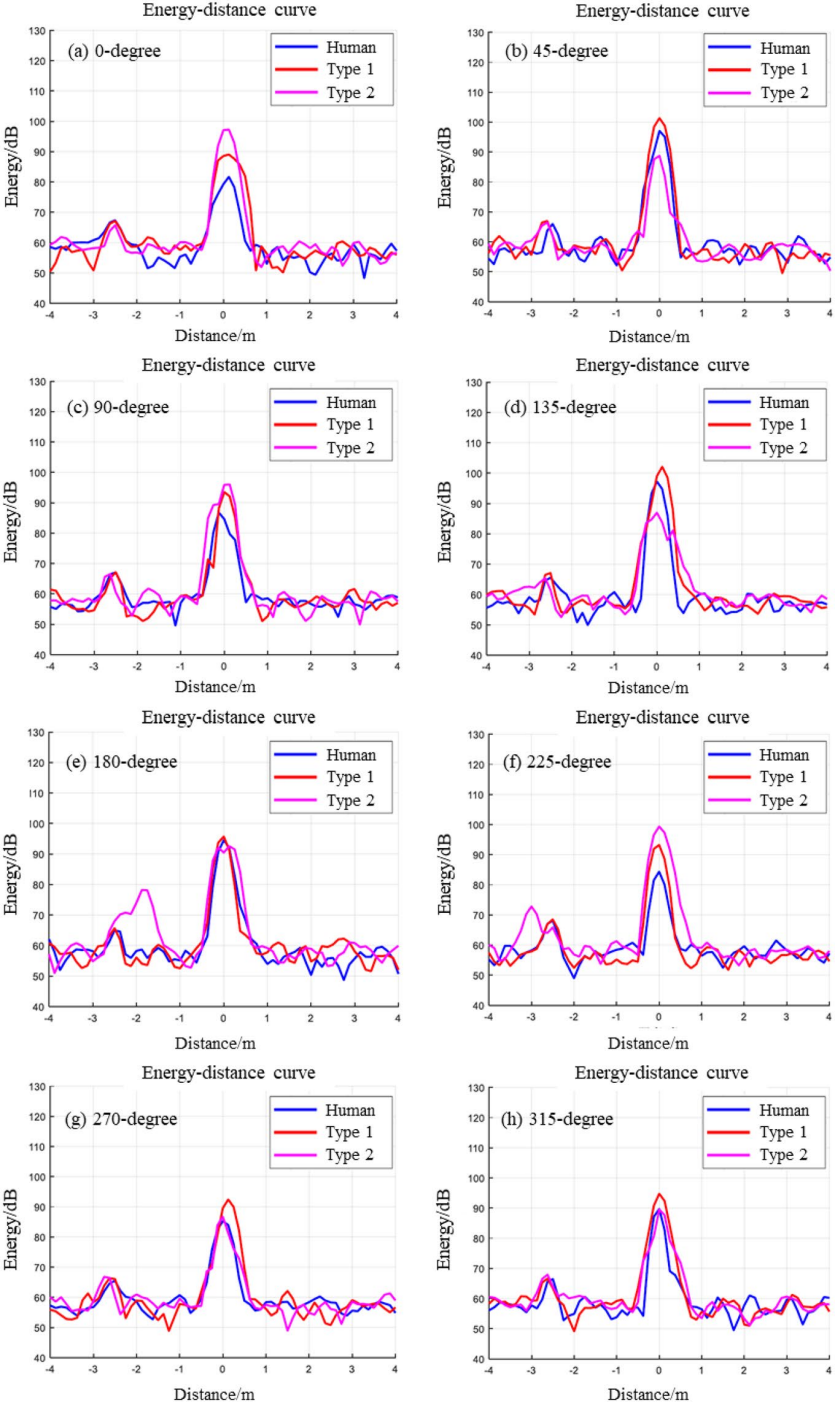
Comparison of energy distance ad different positions.

We conducted the statistical analysis of echo data and calculated the correlation coefficient between the humans and the two types of dummies. The energy relationship between distance and the measured object can be obtained as shown in Fig. 6. In each figure, the blue line represents the echo energy of a real person, the red line represents the echo energy of the Type 1 dummy, and the pink line represents the echo energy of the Type 2 dummy. The correlation coefficients between the energy of human and dummies are calculated by the statistical method, as shown in Table 2. We conducted the statistical analysis of the student’s *t* test to identify whether the difference between each pair of two groups is statistically significant. The results show that in each pair of groups, the difference between them is not significant at the 90% confidence level, suggesting that the results in each group are highly similar.

**Table 2:** Echo energy statistics and correlation coefficient of different angles.

Angle (°)	Correlation coefficient		
	Human & type1 dummy	Human & type2 dummy	Type 1 dummy & Type 2 dummy
0	0.79	0.85	0.75
45	0.87	0.88	0.86
90	0.88	0.89	0.95
135	0.93	0.87	0.84
180	0.90	0.82	0.82
225	0.82	0.85	0.86
270	0.88	0.80	0.90
315	0.86	0.84	0.86

The echo energy of the human is observed to be at its lowest levels at 0 degrees, 90 degrees, and 225 degrees in Fig. 6, while the Type 2 dummy exhibits the highest levels. At angles of 45 degrees, 135 degrees, and 270 degrees, the Type 1dummy demonstrates the highest echo energy. Echo energies for both dummies and humans almost overlap at angles of 180 degrees and 315 degrees. Although there are discernible differences in these echo energies, they are not deemed significantly significant. These disparities primarily arise due to variations in material composition between human bodies and dummies. Different materials possess distinct characteristics pertaining to reflection and scattering properties when exposed to electromagnetic waves from varying angles. Additionally, slight discrepancies may exist in terms of body curvature, joint positioning, or other structural aspects between human bodies and dummies that can influence electromagnetic wave interactions, thereby affecting echo energy outcomes. Furthermore, dissimilarities concerning size and shape between humans and dummies also play a role, as object geometry impacts scattering effects along with reflection phenomena during electromagnetic wave interactions, leading to alterations in echo energy.

From Table 2, it can be observed that only at 0 degrees is there a correlation coefficient of 0.79 between humans and the Type 1 dummy. The obtained correlation coefficient falls within the range of 0.5–0.8, indicating a significant correlation between humans and the Type 1 dummy at this specific angle. For other angles, the correlation coefficients between the Type 1 dummy and humans range from 0.82 to 0.93, suggesting a high level of correlation across different positions. Similarly, for the Type II dummy and humans, the correlation coefficients fall between 0.80 and 0.89, all exceeding or equaling a value of 0.8, thus indicating a strong correlation across all test positions. The variation in these correlation coefficients is closely related to both the angle and cross-sectional area of echo reflection at different angles as well as factors such as clothing material properties for both humans and dummies along with their respective echo reflectivity characteristics.

**Figure 7:**
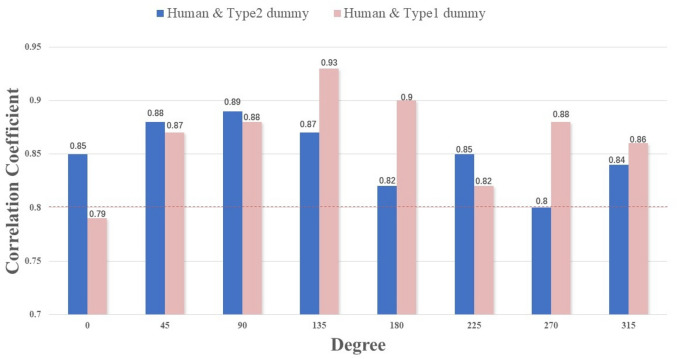
Correlation coefficient comparison chart.

Furthermore, a correlation analysis was conducted to compare the echo energy of the Type I and Type II dummies. At the 0-degree position, a significant correlation coefficient of 0.75 was found between both dummy types, falling within the range of 0.5–0.8. Across different positions, a high degree of correlation ranging from 0.82 to 0.94 was observed between both dummy types. The calculation revealed minimal differences in echo energy between these two dummy types.The correlation coefficients between Type 2 dummies and humans compared with Type 1 dummies and humans are summarized and shown in Fig. 7.

From the data, it can be concluded that the echo energy reflected by the human and dummy is different at different angles, which is caused by the difference in radar cross section (RCS) between the human and dummy at different angles. As shown in Fig. 7, the correlation coefficients of humans and dummies are greater than 0.8, which can draw the conclusion from the correlation theory that there is a high linear correlation between the two variables. In other words, the electromagnetic wave echo characteristics of a dummy and a human are highly associated, which means the dummy can simulate human scenes. At the same time, we also found that the correlation coefficient value of human and Type 2 dummies is higher at 0-degree points, 90-degree points, and 225-degree points, while this value of human and Type 1 dummies is higher at 135-degree points, 180-degree points, 270-degree points, and 315-degree points. From all the above tables of test results, it can be inferred that the correlation between humans and dummies is also very high, and the correlation coefficients are basically greater than or equal to 0.8. Therefore, we can draw the conclusion that the dummies can replace humans in the intelligent and connected vehicle ADAS road test.

The SVM model regression was employed to evaluate the 10 groups of data obtained from each angle test of humans, Type 1 dummies and Type 2 dummies. The SVM model takes experimental factors, such as the type of virtual person and the angle, as input and produces echo energy as output. The evaluation of SVM regression typically employs root mean square error (RMSE) and mean absolute error (MAE) as performance metrics^34^. The RMSE is a metric that calculates the square root of the mean square error between the predicted and actual values of a model. A smaller RMSE indicates a better fit of the model to the actual values. The MAE is a commonly used metric for quantifying the prediction error of a model. It computes the average absolute difference between the predicted values and the actual values. The SVM model prediction involves the division of data into training and test sets. Typically, we allocate 70% of the data as the training set and 30% as the testing set to account for our limited number of model datasets^35^. The classification process employs a programmatic random extraction method. The radial basis function, which is the most commonly employed kernel function in this study, is utilized. The resulting of RMSE and MAE are presented in Table 3.

**Table 3:** Echo energy prediction with SVM model.

Performance	Human		Type 1 dummy		Type 2 dummy	
	RMSE	MAE	RMSE	MAE	RMSE	MAE
Training data set (70% sample)	0.43	5.42	0.52	6.55	0.45	5.74
Testing data set (30% sample)	0.79	9.99	0.90	11.42	0.80	10.12

The results presented in Table 2 demonstrate that the RMSE for the training data are 0.43, 0.52, and 0.45, while the corresponding MAE values are 5.42, 6.55, and 5.74, respectively. Furthermore, it is observed that the RMSE values for the testing set are 0.79, 0.90, and 0.80, with respective MAE values of 9.99, 11.42, and 10.12. The range of RMSE values, from 0.43 to 0.90, suggests a strong degree of fit between the experimental data and the real-world scenario. The MAE values range from 5.42 to 11.42, indicating that the test model exhibits a consistently small average error in predicting the true value. The aforementioned experimental data indicate that there is minimal disparity between the echo energy test results of human subjects and dummies, thereby establishing a high level of reliability in the obtained echo data. The aforementioned experimental data suggest that there is minimal disparity between the echo energy test results of human subjects and dummies, thereby establishing a high level of reliability in the obtained echo data.The findings indicate that the SVM model demonstrates comparable accuracy in predicting ADAS test echo energy, rendering it suitable for practical applications.

## Conclusions

This study evaluated the performance in the experimental tests for ADAS by comparing the data of millimeter-wave radar from humans and dummies models. Millimeter-wave radar is used to collect electromagnetic wave echo characteristics of humans, Type 1 dummies and Type 2 dummies at fixed distances and different angle positions according to the Doppler effect. The conclusion is that the correlation coefficient between the dummies and the real human ranges from 0.75 to 0.93, which means the correlation is high. We also developed the SVM model to predict the echo energy for humans and two dummies in diverse conditions. Reasonable prediction performances were obtained. The MAE is 5.42–11.42 in the training and testing datasets while the RMSE is 0.43–0.90.

The findings of the study can provide useful information and support for practical tests of the ADAS system. First, from the experimental analysis, the study proves that the dummy can replace the humans in the test of the development of the ADAS of the intelligent and connected vehicle. Second, machine learning models such as the SVM can be applied for predicting the echo energy of the millimeter-wave radar which can help find the obstacle more accurately. Finally, the experiment design and the analytical method in this study can provide support for larger-scale ADAS tests for millimeter-wave radar or other types of sensors such as radar.

The paper can be further improved by considering the following future work. In the test, only one middle-aged adult male was selected as the sampling target in the human selection. In the future research, we will increase the number of samples and different environmental scenes as the research object and conduct the in-depth study of the echo characteristics of the dummies to replace the humans more accurately as the environment for the ADAS road test for safety protection. Furthermore, more complex and powerful machine learning models such as deep neural networks^9,36–38^ can be applied to predict the echo energy of millimeter-wave radars in diverse driving environments.

Based on the findings of the research, this study draws the following main conclusions by comparing the millimeter wave radar data in the ADAS experiment test from the human body and the simulation model:

First, the experiment proves that in the development and testing of the ADAS system of intelligent networked vehicles, the dummy model can replace the human body for testing, and the correlation coefficient between the two is between 0.79 and 0.93, indicating a high correlation. Secondly, by using the SVM model, the millimeter-wave radar echo energy of the human body and two simulation models under different conditions are successfully predicted, and reasonable prediction performance is obtained. Most importantly, the findings of this study provide useful information and support for practical testing of ADAS systems. From the experimental analysis, it is not only confirmed that the simulation model can replace the human body for the development and testing of the ADAS system, but it also highlights the application of machine learning models (such as SVM) in the energy prediction of millimeter wave radar echoes, which helps to detect obstacles more accurately. In addition, the experimental design and analysis methods studied provide support for larger-scale ADAS testing with millimeter-wave radars or other sensors such as radar.

## Data Availability

The datasets generated and analysed during the current study are not publicly available due the author is not authorized to share the data. But the datasets are available from the corresponding author on reasonable request.

## References

[1] Dong C, Wang H, Li Y (2019). Route control strategies for autonomous vehicles exiting to off-ramps. IEEE Trans. Intell. Transp. Syst..

[2] Zheng Y, Ran B, Qu X (2019). Cooperative lane changing strategies to improve traffic operation and safety nearby freeway off-ramps in a connected and automated vehicles environment. IEEE Trans. Intell. Transp. Syst..

[3] Han Y, Wang M, He Z (2021). A linear Lagrangian model predictive controller of macro-and micro-variable speed limits to eliminate freeway jam waves. Transp. Res. Part C: Emerg. Technol..

[4] Guo Y, Sayed T, Essa M (2020). Real-time conflict-based Bayesian Tobit models for safety evaluation of signalized intersections. Accid. Anal. Prev..

[5] Li M, Li Z, Xu C (2020). Short-term prediction of safety and operation impacts of lane changes in oscillations with empirical vehicle trajectories. Accid. Anal. Prev..

[6] Wang C, Xie Y, Huang H (2021). A review of surrogate safety measures and their applications in connected and automated vehicles safety modeling. Accid. Anal. Prev..

[7] Galvani M (2019). History and future of driver assistance. IEEE Instrum. Meas. Mag..

[8] Shengmin C (2016). Operation Technology of Intelligent Driving Assistant System.

[9] Zuo Y, Fu X, Liu Z (2021). Short-term forecasts on individual accessibility in bus system based on neural network model. J. Transp. Geogr..

[10] Chen E, Ye Z, Wang C (2019). Subway passenger flow prediction for special events using smart card data. IEEE Trans. Intell. Transp. Syst..

[11] Cao Q, Ren G, Li D (2021). Map matching for sparse automatic vehicle identification data. IEEE Trans. Intell. Transp. Syst..

[12] Zhang B, Chen S, Ma Y (2020). Analysis on spatiotemporal urban mobility based on online car-hailing data. J. Transp. Geogr..

[13] Cao Q, Ren G, Li D (2020). Semi-supervised route choice modeling with sparse Automatic vehicle identification data. Transp. Res. Part C: Emerg. Technol..

[14] Lei D, Chen X, Cheng L (2020). Inferring temporal motifs for travel pattern analysis using large scale smart card data. Transp. Res. Part C: Emerg. Technol..

[15] Xie D, Xu Y, Wang R, Su Z (2018). Obstacle detection and tracking of unmanned vehicle based on 3D laser radar. Automot. Eng..

[16] Lekic V, Babic Z (2019). Automotive radar and camera fusion using generative adversarial networks. Comput. Vis. Image Underst..

[17] Tokoro, S. Automotive application systems of a millimeter-wave radar. Proceedings of Conference on Intelligent Vehicles. IEEE, 1996: 260–265.

[18] Wei T, Han J, Zhou Y, Cao Z (2019). Vehicle target detection with millimeter-wave radar based on machine learning. Bus Coach Technol. Res..

[19] Jing X, Du ZC, Li F (2013). Obstacle detection by Doppler frequency shift. Electron. Sci. Technol..

[20] Ma D, Xiao J, Song X (2020). A back-pressure-based model with fixed phase sequences for traffic signal optimization under oversaturated networks. IEEE Trans. Intell. Transp. Syst..

[21] Ma D, Xiao J, Ma X (2021). A decentralized model predictive traffic signal control method with fixed phase sequence for urban networks. J. Intell. Transp. Syst..

[22] Ma D, Luo X, Jin S (2018). Estimating maximum queue length for traffic lane groups using travel times from video-imaging data. IEEE Intell. Transp. Syst. Mag..

[23] Zhang Q (2019). Research on Object Detection and Tracking Algorithm Based on Millimeter-Wave Radar Sensor.

[24] Zhang Z (2019). Human Body Positioning and Motion State Detection System Based on Radar.

[25] Raz BP (2006). System and Signal.

[26] Nabati, R., Qi, H. C. F. Center-based Radar and Camera Fusion for 3D Object Detection. arXiv 2020. arXiv preprint arXiv:2011.04841.

[27] Shao Y, Zulkefli MAM, Sun Z (2019). Evaluating connected and autonomous vehicles using a hardware-in-the-loop testbed and a living lab. Transp. Res. Part C: Emerg. Technol..

[28] Li L, Wang X, Wang K (2019). Parallel testing of vehicle intelligence via virtual-real interaction. Sci. Robot..

[29] Sudmann A (2019). The Democratization of Artificial Intelligence: Net Politics in the Era of Learning Algorithms.

[30] Roberts RA, Mullis CT (1987). Digital Signal Processing.

[31] Li, Y., Du, L., Liu, H. Analysis of micro-Doppler signatures of moving vehicles by using empirical mode decomposition. Proceedings of 2011 IEEE CIE International Conference on Radar. IEEE, 2011, 1: 600–603.

[32] Ben-Hur A, Horn D, Siegelmann HT (2001). Support vector clustering. J. Mach. Learn. Res..

[33] Drucker, H., Burges, C. J., Kaufman, L. *et al.* Support vector regression machines. Adv. Neural Inform. Process. Syst., 1996, 9.

[34] Cheng, K. Research on Signal Integrity Simulation Technology Based on SVM and PDP. Sichuan University of Science & Engineering,

[35] Kang, W. Design of quantitative trading strategy of stock index futures based on SVM model. Zhongnan University of Economics and Law

[36] Zhang H, Wu Y, Tan H (2020). Understanding and modeling urban mobility dynamics via disentangled representation learning. IEEE Trans. Intell. Transp. Syst..

[37] Liu Y, Lyu C, Liu X (2020). Automatic feature engineering for bus passenger flow prediction based on modular convolutional neural network. IEEE Trans. Intell. Transp. Syst..

[38] Chen X, Li Z, Yang Y (2020). High-resolution vehicle trajectory extraction and denoising from aerial videos. IEEE Trans. Intell. Transp. Syst..

